# Enhancement of photocatalytic H_2_ evolution of eosin Y-sensitized reduced graphene oxide through a simple photoreaction

**DOI:** 10.3762/bjnano.5.92

**Published:** 2014-06-06

**Authors:** Weiying Zhang, Yuexiang Li, Shaoqin Peng, Xiang Cai

**Affiliations:** 1 Department of Chemistry, Nanchang University, Nanchang 330031, China

**Keywords:** eosin Y sensitization, graphene oxide, H_2_ evolution, photocatalysis, photoreduction, sp^2^ conjugated domains

## Abstract

A graphene oxide (GO) solution was irradiated by a Xenon lamp to form reduced graphene oxide (RGO). After irradiation, the epoxy, the carbonyl and the hydroxy groups are gradually removed from GO, resulting in an increase of sp^2^ π-conjugated domains and defect carbons with holes for the formed RGO. The RGO conductivity increases due to the restoration of sp^2^ π-conjugated domains. The photocatalytic activity of EY-RGO/Pt for hydrogen evolution was investigated with eosin Y (EY) as a sensitizer of the RGO and Pt as a co-catalyst. When the irradiation time is increased from 0 to 24 h the activity rises, and then reaches a plateau. Under optimum conditions (pH 10.0, 5.0 × 10^−4^ mol L^−1^ EY, 10 μg mL^−1^ RGO), the maximal apparent quantum yield (AQY) of EY-RGO24/Pt for hydrogen evolution rises up to 12.9% under visible light irradiation (λ ≥ 420 nm), and 23.4% under monochromatic light irradiation at 520 nm. Fluorescence spectra and transient absorption decay spectra of the EY-sensitized RGO confirm that the electron transfer ability of RGO increases with increasing irradiation time. The adsorption quantity of EY on the surface of RGO enhances, too. The two factors ultimately result in an enhancement of the photocatalytic hydrogen evolution over EY-RGO/Pt with increasing irradiation time. A possible mechanism is discussed.

## Introduction

Hydrogen is an efficient and green energy carrier. Photocatalytic water splitting into hydrogen by means of solar energy and semiconductor photocatalysts is a environmentally friendly way to produce storable energy [1–4]. In order to enhance the activity of photocatalysts for hydrogen evolution, various graphene-based composite photocatalysts, such as graphene/TiO_2_ composite and graphene/ZnO composite, have recently been reported [5–8]. Kim et al. [8] have reported that two graphene/TiO_2_ composites, a nanographene shell on a TiO_2_ core and TiO_2_ nanoparticles on a graphene sheet, exhibit a higher photocatalytic H_2_ evolution than TiO_2_ under UV irradiation. This can be attributed to an efficient electron transfer from TiO_2_ to graphene [9–10]. Interestingly, single reduced graphene oxide itself (RGO) shows a higher activity as a semiconductor under UV irradiation [11–12]. Yeh et al. [12] reported that RGO sheets with in situ photoreduced platinum displayed a high activity for hydrogen evolution from an aqueous methanol solution. However, the RGO exhibits a very low photocatalytic activity under visible light irradiation.

Eosin Y (EY), a xanthene dye, is a very good sensitizer [13–18]. EY has been used to sensitize various matrixes such as TiO_2_ [13], Na_2_Ti_2_O_4_(OH)_2_ nanotubes [14], g-C_3_N_4_ [15], and α-[AlSiW_11_(H_2_O)O_39_]^5−^ [18], and the sensitized photocatalysts are characterized by a high activity for H_2_ evolution under visible light irradiation. Recently, to improve the photocatalytic activity for hydrogen evolution in the visible light region, EY has been employed to sensitize RGO, and the sensitized photocatalyst displays an increased photoactivity for hydrogen evolution [19–21]. Mou et al. [19] found that the photocatalytic activity of EY-RGO for hydrogen evolution was superior to that of EY-GO. However, the activity of the former was still very low. Min and Lu [20] demonstrated a successful example of enhancing H_2_ evolution activity by using RGO as an efficient electron relay between the photoexcited EY and the loaded Pt co-catalyst, which shows an AQY of 4.15% under visible light irradiation. In these works, RGO was obtained by a chemical reduction of GO with hydrazine or sodium borohydride as a reductant.

Graphene, an atom-thick two-dimensional (2D) sheet of sp^2^-hybrized carbon, has received tremendous research interests based on its extraordinary electronic, thermal, optical and excellent electron transport properties [21–22]. Graphene can be easily obtained by reducing graphene oxide (GO), which is a cheap and scalable preparation method [23–26]. The GO contains not only hydroxy and epoxy groups in the 2D sheet, but also carbonyl and carboxyl groups at the edges of the sheet [27–28]. The oxygen-containing groups in the sheet break the sp^2^ π-conjugation, leading to the formation of oxidized aliphatic six-membered rings with sp^3^-hybridization in the GO layer. As a result, the conductivity of GO decreases greatly compared with that of graphene. Amongst various methods for the reduction of GO to form RGO, photoreaction (photoreduction) is “green” without any toxic chemical reagents. Moreover, it is easy to control the degree of reduction by applying different UV irradiation times [29–32]. The RGO prepared by UV irradiation is of high dispersion, can be stored for a long time without getting aggregated, and exists in the quasi homogeneous form [33–34].

In this work, we prepared RGO starting from an aqueous GO solution by controlling UV irradiation time. The RGO solution and its powder were denoted as RGO*x* and RGO*x*-p, respectively, where *x* (in hours) represents the particular UV irradiation time. The photocatalytic activity of EY-sensitized RGO*x* was investigated by using Pt as a co-catalyst and trimethylamine (TMA) as a sacrificial electron donor. To the best of our knowledge, studies on the effect of irradiation time on the performance of RGO*x* for the dye sensitized H_2_ generation have not been reported so far. The sensitization activity for hydrogen evolution under visible light illumination is much higher than that of EY-RGO/Pt produced by chemical reduction methods in the literature [19–20]. A possible mechanism is discussed.

## Results and Discussion

### The effect of irradiation time on the performance of RGO*x*

**Figure 1:**
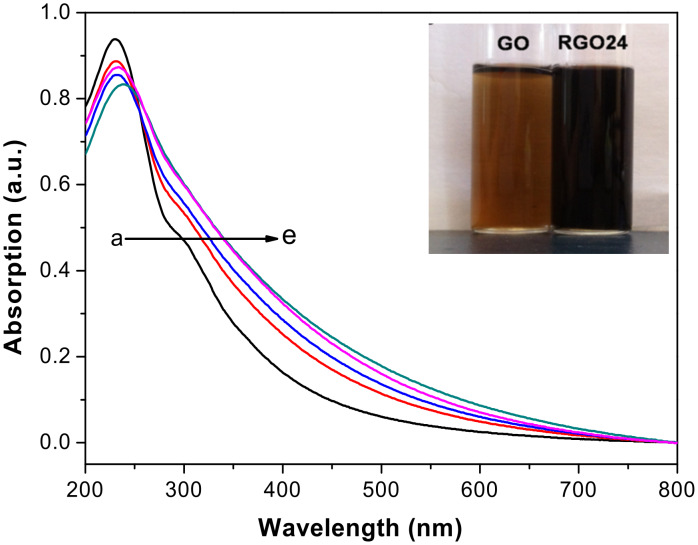
UV–vis spectra of (a) GO, (b) RGO4, (c) RGO12, (d) RGO24, and (e) RGO36 solution (20 μg mL^−1^). The inset is an image of GO and RGO24.

**Scheme 1:**
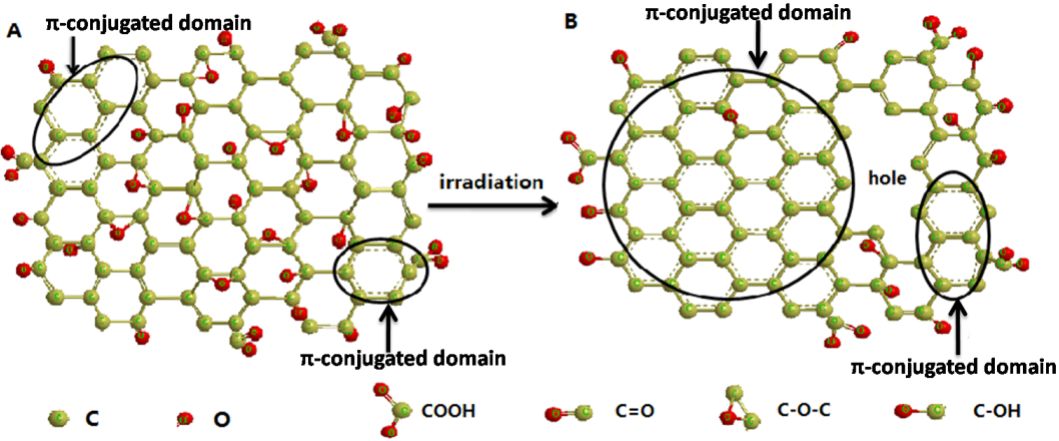
Schematic diagram of the reduction of GO by irradiation.

Figure 1 shows UV–vis spectra of GO and RGO*x* solution. The peak at 232 nm is due to the C=C bond in an aromatic ring [35], whereas the broad shoulder peak at 291 nm can be assigned to C=O [36]. The absorption over 291 nm is expected to be caused by the conjugated fused ring plane [37]. An increase of the irradiation time from 0 to 24 h entails an increase of the absorption strength of the RGO*x* solution over 291 nm and a red-shift of the absorption. The absorption of RGO36 is close to that of RGO24, indicating that the deoxygenation reaction takes place slowly at that stage [38]. This can be attributed to an enhancement of π-conjugated sp^2^ domains (restoration of sp^2^ π-conjugated network) by the removal of oxygen-containing groups whose carbon atoms are sp^3^ hybridized, and a decrease of sp^3^ domains (see Mechanism section, Scheme 1). The inset of Figure 1 shows a picture of GO and RGO24. The GO dispersion is yellow–brown and semitransparent, gradually changes to dark brown after irradiation (not shown here), and finally turns black and opaque after 24 h of irradiation. This indicates an increase of the degree of sp^2^ conjugation by a regeneration of the sp^2^ π-conjugated network [35].

**Figure 2:**
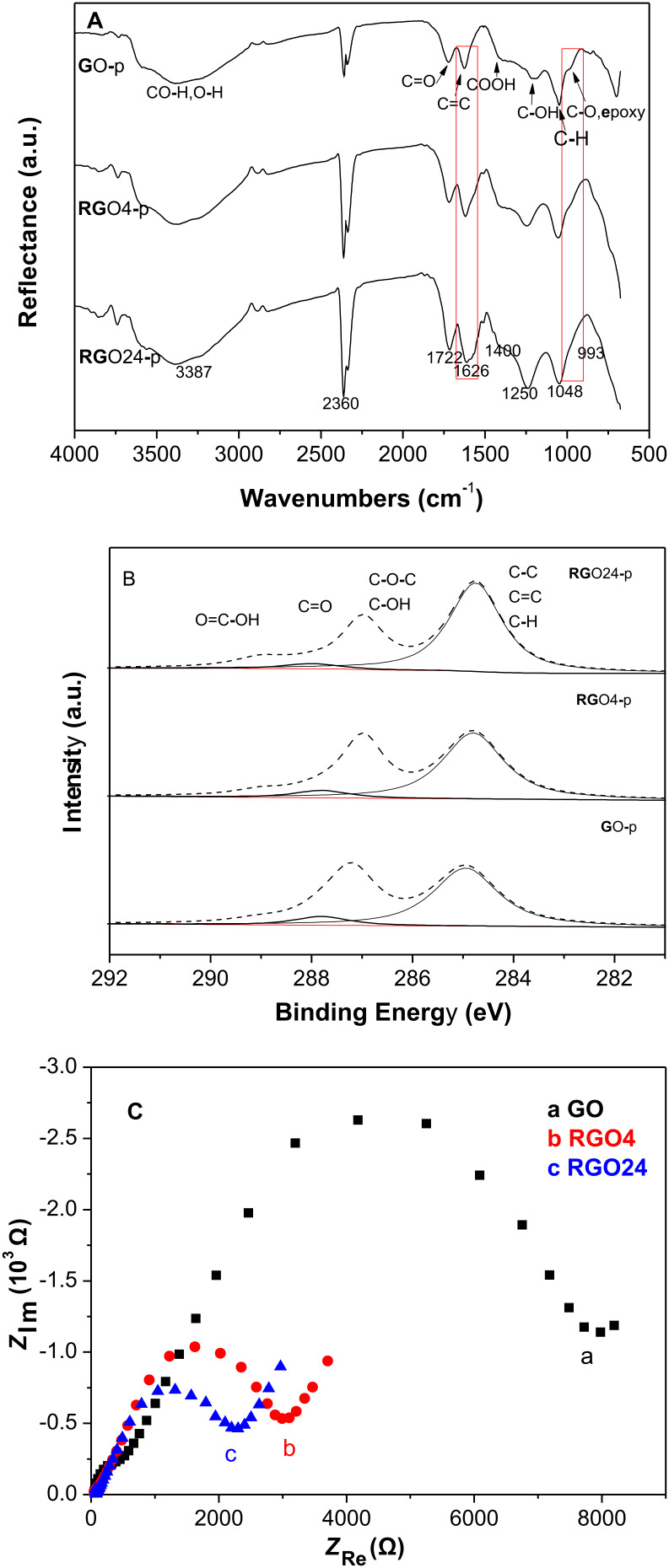
(A) ATR-IR spectra of GO-p, RGO4-p and RGO24-p, (B) XPS spectra of C1s for GO-p, RGO4-p and RGO24-p, and (C) Nyquist diagrams of GO, RGO4 and RGO24.

Figure 2A displays ATR-IR spectra of GO-p, RGO4-p and RGO24-p. The peak intensity at 1626 cm^−1^, which can be assigned to C=C [39], increases with irradiation time, whereas the epoxy C–O peak at 993 cm^−1^ [12] disappears after irradiation of 24 h. These suggest that epoxy C–O–C has been reduced to produce sp^2^ carbons [40]. Figure 2B shows XPS spectra of C1s for GO-p, RGO4-p and RGO24-p. The deconvoluted peaks centered in a binding energy range of 284.8–285.0 eV and 287–287.2 eV are attributed to C–C, C=C, and C–H bonds, and C–O bonds (C–O–C and C–OH), respectively [30]. The deconvoluted peaks at binding energy of 287.8 eV and 289.0 eV belongs to C=O and O=C–OH respectively [41]. The peak intensities of epoxy C–O–C and C=O decrease with increasing irradiation time, which suggests that most of epoxy C–O–C and C=O are removed to produce sp^2^ domains [40]. Clearly, the peak intensities of C–C, C=C, and C–H bonds increase. As shown in Table 1, the O/C atomic ratio decreases from 0.33 in the GO sample to 0.26 after 24 h of photoreaction. This further indicates the restoration of the sp^2^ π-conjugated network for RGO after the photoreaction. Due to restoration of the sp^2^ π-conjugated network in RGO*x*, its conductivity is expected to increase [42]. To verify this enhancement, the electrochemical impedance spectroscopy (EIS) of GO, RGO4 and RGO24 were measured. Figure 2C shows the Nyquist diagrams for GO, RGO4 and RGO24. The semicircles correspond to the charge transfer resistance (*R*_CT_) [43] and become smaller and smaller after irradiation. This suggests that their conductivity order is RGO24 > RGO4 > GO, which is due to the increase of the aromatic ring plane, more specifically, the increase of sp^2^ π-conjugated domains [44].

**Table 1:** Peak area ratios of oxygen-containing bonds to CC bonds and O/C ratio obtained from Figure 2B.

sample	A_C–O_/A_CC_	A_C=O_/A_CC_	A_OCOH_/A_CC_	O/C

GO	0.69	0.13	0.05	0.33
RGO4	0.59	0.10	0.07	0.30
RGO24	0.42	0.06	0.08	0.26

### The interaction between EY and GO/RGO*x*

**Figure 3:**
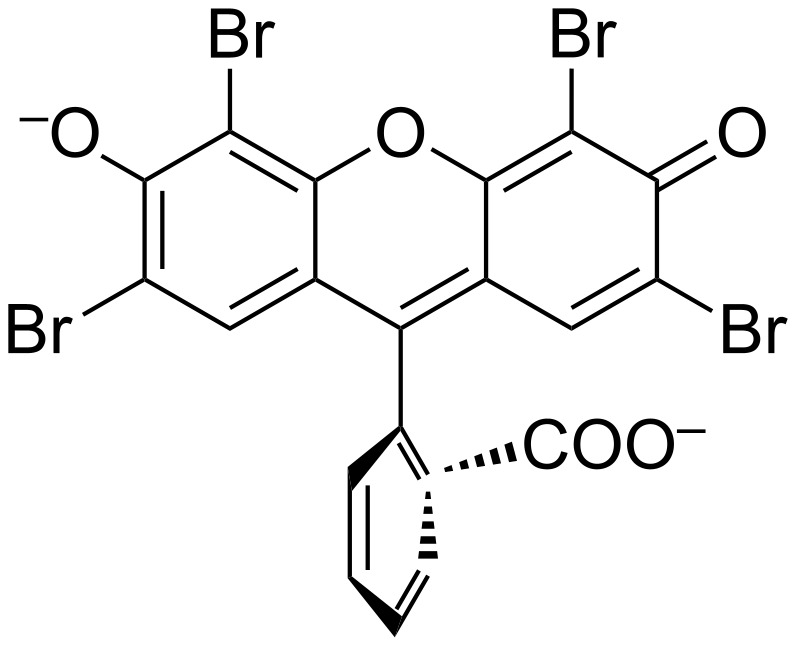
Chemical structure of EY.

The chemical structure of EY is shown in Figure 3. The benzoate ring is perpendicular to the xanthenes moiety. The main interaction between EY and graphene is through π–π stacking [19–21] of the xanthene moiety (the fused ring) of EY with sp^2^ π-conjugated domains of graphene.

Table 2 lists the amount of adsorbed EY on the surface of RGO*x*. Before the irradiation, the adsorption amount of EY on the GO surface is 11.7 μmol g^−1^. After irradiation of GO for 4, 12 and 24 h, the adsorption amount increases to 15.0, 32.7 and 74.3 μmol g^−1^, respectively. This is because the sp^2^ domains of RGO*x* increase with the reaction time (see Mechanism section, Scheme 1). As a result, the π–π stacking interaction between EY and RGO becomes stronger.

**Table 2:** Adsorbed amount of EY on the surface of GO or RGOx.

sample	GO	RGO4	RGO12	RGO24

amount of adsorbed EY (μmol g^−1^)	11.7	15.0	32. 7	74.3

**Figure 4:**
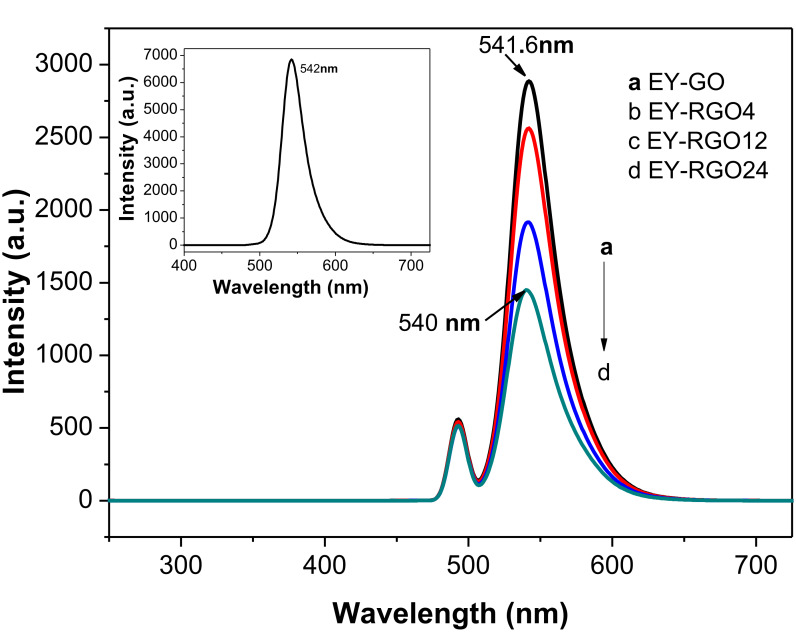
Fluorescence spectra of EY-RGO*x* in TMA solution. The inset shows the fluorescence spectrum of EY in TMA solution. Conditions: 30 μg mL^−1^ GO or RGO*x*; 1.0 × 10^−5^ mol L^−1^ EY; 7.7 × 10^−2^ mol L^−1^ TMA.

To further confirm the interaction between EY and GO/RGO*x*, the fluorescence spectra of the mixture of EY and GO or RGO*x* were measured, as shown in Figure 4. The inset of Figure 4 displays a strong fluorescence peak of EY (about 6.8 × 10^3^ a.u.) at 542 nm. This can be attributed to the large visible light absorption by its conjugated xanthenes structure and the strong recombination of photo-excited electron–hole pairs. When GO is added to the EY solution, the fluorescence intensity of EY at 541.6 nm sharply declines to about 2.8 × 10^3^ a.u. The addition of RGO24 results in a great fluorescence quenching of EY (Figure 4d,), the emission peak intensity decreases to about 1.4 × 10^3^ a.u., and the fluorescence peak shifts from 541.6 to 540.0 nm. This is because the number of sp^2^ π-conjugated domains (larger adsorption amount for EY) and the conductivity of RGO*x* increase with longer irradiation times, thereby enhancing the ability for a photo-induced electron transfer from the excited dye molecules to RGO*x*. The slight blue shift suggests that an intermolecular π-π stacking interaction between RGO24 and EY is stronger than the interaction between GO and EY [20].

**Figure 5:**
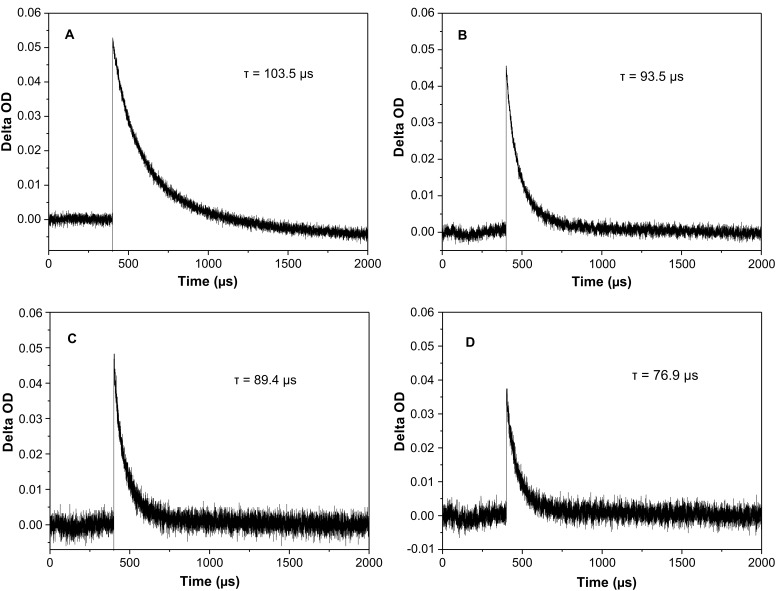
Transient absorption decay of ^3^EY^*^ followed at 580 nm for (A) EY, (B) EY−GO, (C) EY−RGO4, and (D) EY−RGO24 under pulse irradiation of 532 nm. Conditions: 30 μg mL^−1^ GO or RGO*x*; 2.0 × 10^−5^ mol L^−1^ EY.

To further confirm the increased ability to transfer electrons between RGO*x* and EY, transient absorption decay spectra of ^3^EY^*^ at 580 nm in EY-RGO*x* and EY-GO systems were measured on a laser flash photolysis spectrometer (Figure 5). It is well-known that EY molecules are excited to the singlet excited state (^1^EY^*^) which is of short-life, and then produce long-lived triplet excited states (^3^EY^*^) by inter-system crossing [45–46]. ^3^EY^*^ has an absorption peak below 580 nm [47]. In the absence of GO or RGO*x*, the lifetime of ^3^EY^*^ is 103.5 μs, while in the presence of GO, the lifetime of ^3^EY^*^ is reduced to 93.5 μs. The addition of RGO4 and RGO24 yields a declined lifetime of ^3^EY^*^ to 89.4 and 79.6 μs, respectively. This result confirms that RGO*x* can transfer the electron of ^3^EY^*^ more effectively than GO [16]. The ability to transfer electrons in decreasing order is RGO24 > RGO4 > GO, which is in accordance with the result of the fluorescence spectra.

### Photocatalytic H_2_ evolution

**Figure 6:**
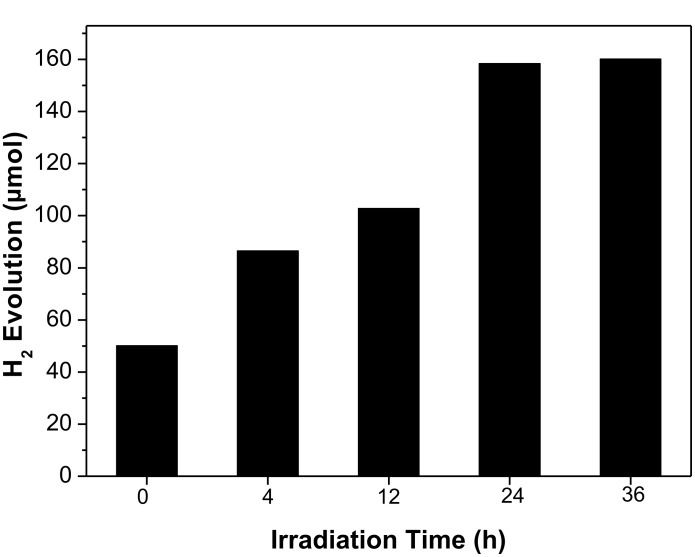
Photocatalytic H_2_ evolution of EY sensitized GO and RGO*x*. Conditions: 30 μg mL^−1^ GO or RGO*x*; 1.45 × 10^−4^ mol L^−1^ EY; 4.6 × 10^−6^ mol L^−1^ H_2_PtCl_6_; 7.7 × 10^−2^ mol L^−1^ TMA, pH 11.9; irradiation 2 h.

Figure 6 shows the photocatalytic H_2_ evolution of EY-sensitized GO and RGO*x* under visible light irradiation. The amount of H_2_ evolution increases with an increase of the irradiation time from 0 to 24 h, and then keeps almost unchanged. In the absence of GO or RGO*x*, the photocatalytic activity of the EY–Pt system is 21.5 μmol, whereas in the absence of EY, no hydrogen is produced from the RGO24/Pt system. This suggests that the visible light activity is comes from the EY sensitization. When GO is added to the EY solution, the photocatalytic activity is 50.1 μmol. When RGO24 is added, the H_2_ evolution reaches 156.8 μmol. The activity increases by a factor of 3.1 compared to the activity of EY-GO/Pt. This result can be attributed to an enhancement of the adsorption quantity of EY on the surface of RGO*x* and the increased electron transfer ability (Table 2 and Figures 2,4,5). However, further irradiation of GO does not lead significant changes of the photocatalytic activity. This may be attributed to a slow increase of the sp^2^ domains of RGO*x* after an irradiation time of over 24 h.

**Figure 7:**
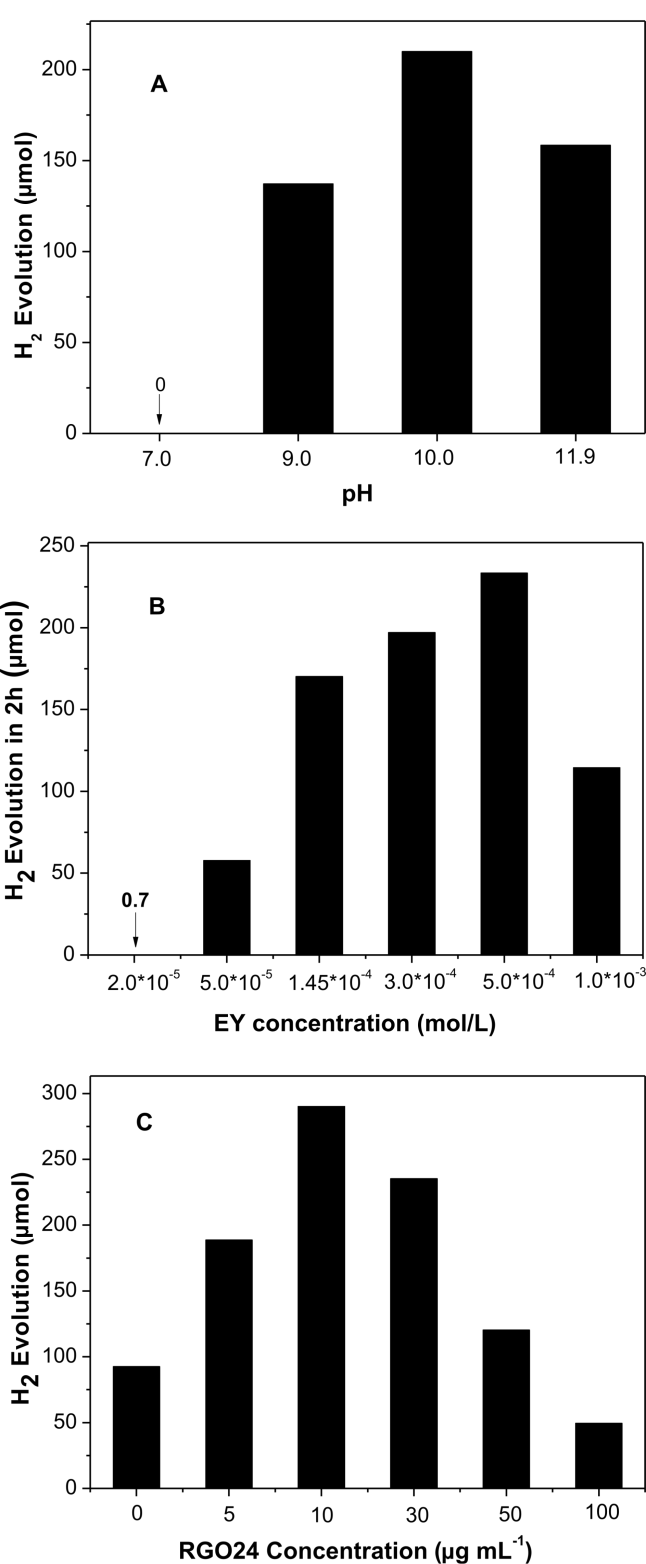
(A) The effect of the pH value on the photocatalytic activity of EY-RGO24/Pt. Conditions: 30 μg mL^−1^ RGO24; 1.45 × 10^−4^ mol L^−1^ EY; 4.6 × 10^−6^ mol L^−1^ H_2_PtCl_6_; 7.7 × 10^−2^ mol L^−1^ TMA; irradiation 2 h. (B) The effect of the EY concentration on the photocatalytic H_2_ evolution over EY-RGO24/Pt. Conditions: 30 μg mL^−1^ RGO24; 4.6 × 10^−6^ mol L^−1^ H_2_PtCl_6_; 7.7 × 10^−2^ mol L^−1^ TMA, pH 10.0; irradiation 2 h. (C) The effect of the RGO24 concentration on the photocatalytic H_2_ evolution over EY-RGO24/Pt. Conditions: 5.0 × 10^−4^ mol L^−1^ EY; 4.6 × 10^−6^ mol L^−1^ H_2_PtCl_6_; 7.7 × 10^−2^ mol L^−1^ TMA, pH 10.0; irradiation 2 h.

We also investigated the effects of the pH value, EY and RGO24 concentration on the photocatalytic activity for hydrogen evolution over EY-RGO24/Pt. Figure 7A shows that the pH value has a remarkable effect on the photocatalytic activity in the presence of TMA as a sacrificial donor. The hydrogen evolution of EY-RGO24/Pt increases with a rise of the pH from 7.0 to 10.0, and then decreases starting at 11.9 (nature pH). When the pH value of the TMA solution is 7.0, no hydrogen evolution is observed, because TMA (p*K*_b_ = 4.22) is completely protonated and TMAH^+^ cannot act as an effective electron donor [48]. With the pH value increasing, more and more TMA species exists in its molecular form. Thus, the activity increases with the pH value and reaches a maximal value at pH 10.0. However, over pH 10.0, the activity decreases. This is caused by the increasing negativity of the redox potential of H^+^/H_2_, which is disadvantageous for an efﬁcient generation of hydrogen [49].

Figure 7B displays the effect of the concentration of EY on the photocatalytic activity. The activity increases with increasing concentrations of EY. The maximal activity is at 5.0 × 10^−4^ mol L^−1^ and then decreases with higher concentrations. When the concentration of EY is 2.0 × 10^−5^ mol L^−1^, the amount of generated hydrogen in 2 h is very low, only 0.7 μmol. This may be due to the deceleration of the light absorption of EY by RGO24, which results in the formation of few photo-excited electrons at low concentrations of dye. When the EY concentration increases to 5.0 × 10^−4^ mol L^−1^, more and more EY molecules adsorb at RGO24, which can effectively absorb photons and transfer photo-induced electrons into the sp^2^ domains of RGO24 for hydrogen evolution. Nevertheless, with a further increase of the concentration of EY, more and more free EY molecules are in solution. These free dye molecules cannot effectively transfer their photo-excited electrons to RGO. Moreover, excess EY in solution may not only screen the light absorption of EY-RGO but also produce self-quenching, which greatly decreases the utilization efficiency of the incident light [20,50]. Thus, the photocatalytic activity decreases at 1.0 × 10^−3^ mol L^−1^ EY.

Figure 7C shows the influence of the concentration of RGO24 on the activity of hydrogen evolution. The activity enhances with an increase of the RGO24 concentration and then declines. The optimal concentration of RGO24 is 10 μg mL^−1^, and the corresponding activity for hydrogen evolution is 290 μmol. Its calculated AQY reaches 12.9%, which is much higher than the reported AQY of EY-sensitized RGO obtained by sodium borohydride reduction [20]. The activity decreases notably at higher RGO24 concentrations, which is ascribed to a strong visible light absorption of RGO24 (Figure 1), which shields the absorption of EY.

**Figure 8:**
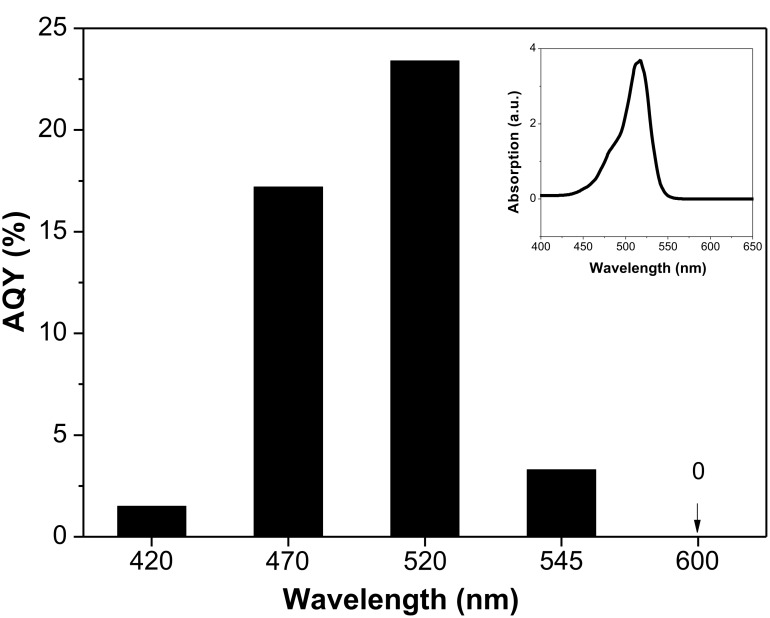
AQY of the EY-RGO24/Pt photocatalyst plotted as a function of the wavelength of the incident light. Conditions: 10 μg mL^−1^ RGO24; 5.0 × 10^−4^ mol L^−1^ EY; 4.6 × 10^−6^ mol L^−1^ H_2_PtCl_6_; 7.7 × 10^−2^ mol L^−1^ TMA, pH 10.0; irradiation 2 h. The inset is the UV–vis absorption spectrum of EY in TMA solution.

The wavelength dependence of the photocatalytic H_2_ evolution of EY-RGO24/Pt was investigated. Figure 8 displays the AQY for EY-RGO24/Pt as a function of the incident light wavelength. AQY increases with increasing wavelength, the highest AQY is 23.4% at 520 nm, and then it decreases. This corresponds with the absorption wavelength of EY in TMA solution (inset of Figure 8).

### Mechanism

When GO is irradiated by UV light, holes h^+^ (in HOMO) and electrons e^−^ (in LUMO) are produced in its π-conjugated domains due to π–π* band excitation [35]. These holes (h^+^) and electrons (e^−^) react with the oxygen-containing groups of GO sheets, e.g., the epoxy groups. The reactions can be expressed as follows [51]:

[1]
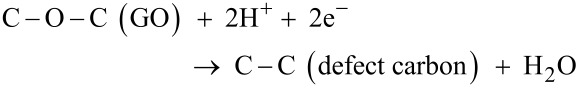


[2]
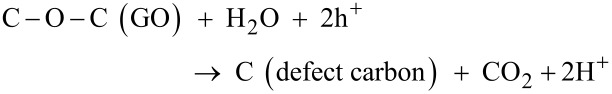


Similarly, photoreactions of the hydroxy group of the GO can be described by the following reactions:

[3]
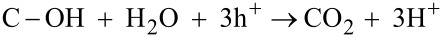


[4]
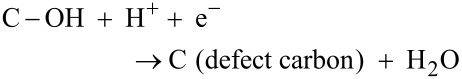


For the C=O and O=C–OH groups of GO, similar reactions take place to form defect carbon atoms. In our irradiation system for GO, due to the presence of O_2_ (see Experimental section), the solved O_2_ would trap the electrons:

[5]
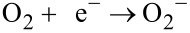


The formed O_2_^−^ is a strong oxidant which can also oxidize the oxygen-containing groups to form CO_2_ and defect carbon atoms. However, at a later stage of the reaction, the oxidation action decreases, as more and more O_2_ is consumed in the closed reaction system.

**Figure 9:**
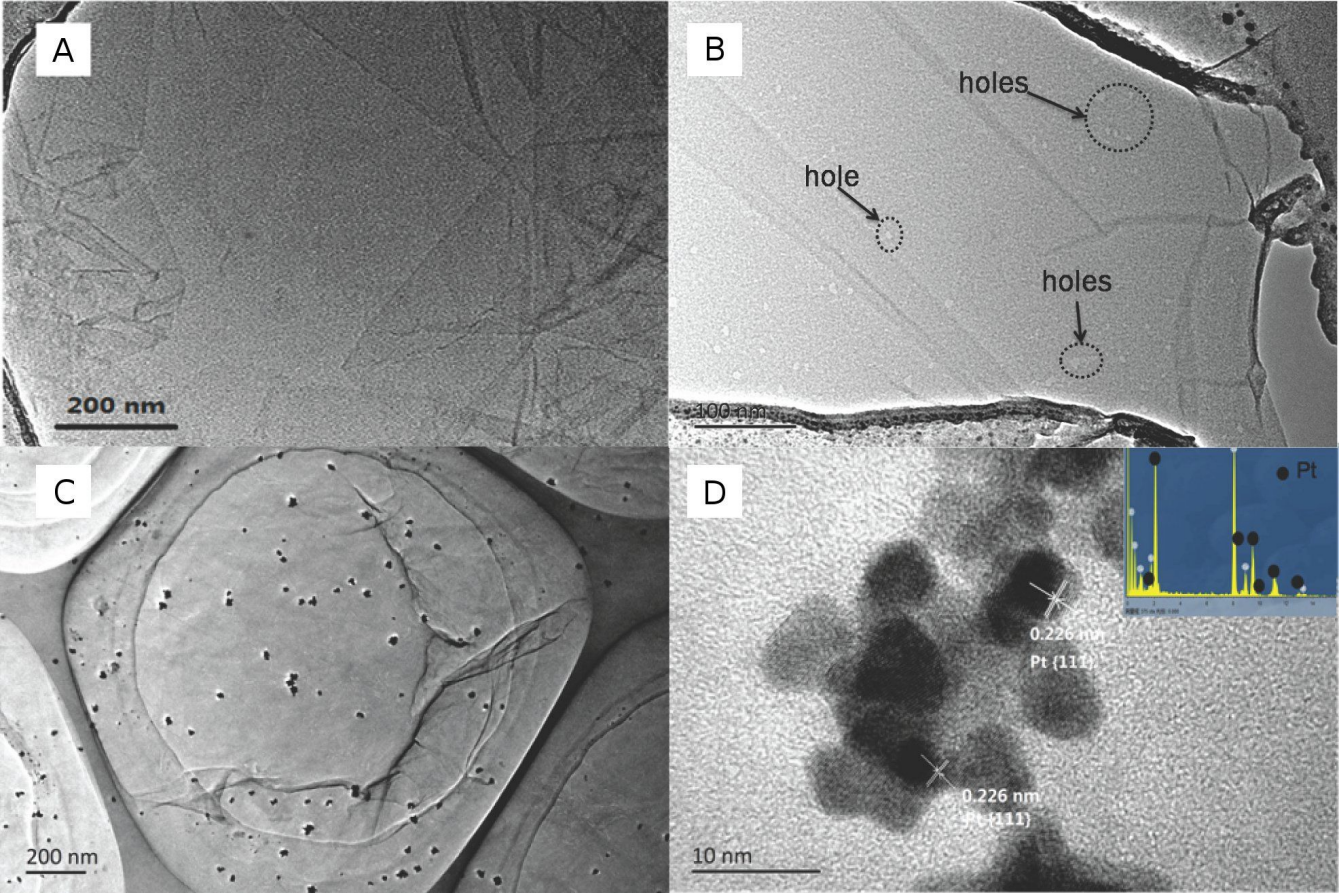
TEM images of GO (A), RGO24 (B), RGO24 with deposited Pt (C), and HRTEM image of deposited Pt (D). The inset of Figure 9D is the EDS spectrum.

The formed defect carbons (radicals) are active, and are expected to react to form C=C. As shown in Scheme 1, π-conjugated domains extend, which is consistent with the results shown in Figure 1 and Figure 2. At the same time , many holes in RGO*x* sheets occur caused by the oxidation of the holes and O_2_^−^ produced by the UV excitation. Figure 9A and Figure 9B show the morphology change of GO before and after the photoreaction. Before the irradiation, GO are complete sheets except for a few wrinkles. After the irradiation, many small holes occur in the RGO24 sheet (Figure 9B), which is consistent with the model shown in Scheme 1. The model is similar to the one reported in [51].

The ferromagnetic properties [51] of RGO obtained by a photoreaction and its paramagnetic resonance (EPR) spectra [38] indicate that there are some radicals or defect carbons at the zigzag hole edges of the RGO. The defect carbons or radicals are stable due to larger π-conjugated domains, which are expected to exist for a long time at room temperature [38,52].

When EY adsorbed at RGO*x* absorbs the visible light, the excited EY^*^ forms by transferring its HOMO electron to the LUMO. The formed EY^*^ injects its electron into the RGO*x* to produce EY^+^. The electron can be transferred to the radicals or defect carbons of RGO*x* via the π-conjugated domains (higher conductivity) to form RGO*x*^−^ ions. Then, EY^+^ is transformed into EY by the electron donor TMA. These processes can be described by the following reactions.

[6]
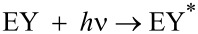


[7]
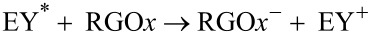


[8]
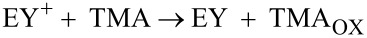


The formed RGO*x*^−^ can reduce PtCl_6_^2−^ at the zigzag edges of the RGO*x*.

[9]
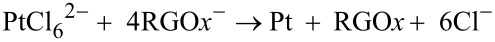


Figure 9C shows the Pt nanoparticles deposited on the surface of RGO24 by an in situ photoreduction of H_2_PtCl_6_ with EY sensitization. It clearly displays the uniform Pt aggregated nanograins with a diameter of 24–30 nm. High-resolution TEM (Figure 9D) shows that the Pt nanograins consists of small Pt nanoparticles with a diameter of about 5 nm. The lattice spacing of 0.226 nm could be indexed to the {111} planes of Pt. After the Pt deposition, the electron from the excited EY can transfer to the radicals or defect carbons to form RGO*x*^−^ ions, which would be trapped by the deposited Pt to reduce water into H_2_.

[10]
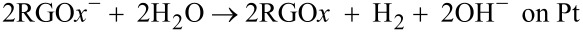


**Scheme 2:**
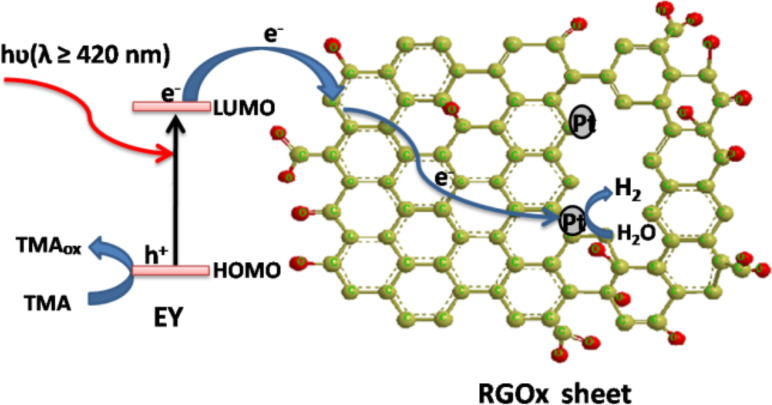
Proposed mechanism for the photocatalytic hydrogen evolution of a EY-RGO*x*/Pt system under visible light irradiation.

The possible mechanism for the photocatalytic H_2_ evolution in a EY-RGO*x*/Pt system is described by Scheme 2.

## Conclusion

In summary, RGO*x* was prepared by a simple photoreaction through controlling irradiation time. After the irradiation, the epoxy, carbonyl and hydroxy groups of GO are gradually removed, sp^2^ π-conjugated domains increase, and the formed RGO sheets have holes. The conductivity of RGO*x* and the adsorption amount of EY on the surface of RGO*x* increase with the irradiation time. The two factors lead to the enhancement of the photocatalytic hydrogen evolution over EY-RGO/Pt with increasing irradiation time. The maximal apparent quantum yield of EY-RGO24/Pt for hydrogen evolution is up to 12.9% under visible light irradiation (λ ≥ 420 nm), and 23.4% under monochromatic light irradiation at 520 nm.

## Experimental

### Preparation of GO

All the reagents were of analytical grade except EY (bioreagent) and were used without further purification. Graphite oxide was synthesized from natural flake graphite powder (Sinopharm Chemical Reagent Co. Ltd) by the modified Hummers method [53–54]. In a typical method, natural graphite (2 g), NaNO_3_ (1 g), and H_2_SO_4_ (46 mL) were mixed in an ice bath, then KMnO_4_ (6 g) was slowly added to this mixture, and was stirred continuously for 30 min. Then the resulting mixture was heated to a temperature of 35 °C and was stirred for 2 h. 90 mL of distilled water was added slowly to the mixture for 20 min. Then the mixture was rapidly heated to 98 °C and kept stirring for 15 min. 144 mL of distilled water and 20 mL of H_2_O_2_ were added to the mixture. After the reaction, the obtained yellow-brown dispersion of graphite oxide was washed with 5% HCl and water until pH 5 and dried in an oven at 60 °C. 0.5 g of graphite oxide powder was added into 1 L of distilled water, and the dispersion was treated with ultrasound (KQ-800KDB, KunShan Ultrasonic Instrument Co. Ltd) for 2 h until the solution became clear to obtain a graphene oxide (GO) solution.

### Photoreaction of GO

100 mL of GO solution (0.5 mg mL^−1^) in a sealed Pyrex flask with a flat window was irradiated with a Xenon lamp (XQ350, ShangHai LanSheng Electronics Co. Ltd.). The headspace of the flask is air. The irradiation time was 4, 12, 24 and 36 h, respectively. The obtained reduced graphene oxide solution is denoted as RGO*x*, where *x* represents the reaction time in hours. In order to characterize the performance of RGO*x*, its powder (denoted as RGO*x*-p) was obtained by centrifuging at 12000 rpm for 30 min and drying at 120 °C.

### Characterization methods

An X-ray photoelectron spectrometer (XPS) was used to analyze GO and RGO*x* on an ESCALAB250xi equipped with a Mg Kα X-ray source. The C1s peak set at 284.8 eV was used as an internal reference for the absolute binding energy. Attenuated total reflection infrared (ATR-IR) spectra were recorded on a FTIR Nicolet 5700 spectrometer equipped with a ZnSe crystal horizontal ATR unit. UV–vis absorption spectra were measured on a Hitachi U-3310 spectrophotometer with distilled water for reference. The fluorescence spectra were measured on a Hitachi F-7000 fluorescence spectrophotometer. The transmission electron microscopy (TEM) and high-resolution TEM (HRTEM) images were taken on a JEOL JEM-2010 (TEM) equipped with an energy dispersive spectrometer (EDS).

Electrochemical impedance spectroscopy (EIS) was measured on an IVIUMSTAT electrochemical workstation (Netherlands). The electrochemical experiments were performed in a 3-compartment cell with a glassy carbon electrode (diameter 2 mm) as the working electrode, a platinum wire as the counter electrode, and an Ag/AgCl electrode as the reference electrode. The electrolyte was a solution of 0.1 M phosphate buffer solution (PBS, pH 7), 0.1 M KCl, 10 mM K_3_Fe(CN)_6_ and 10 mM K_4_Fe(CN)_6_.

Adsorption amounts of EY on GO and RGO*x* were measured as follows: 6 mL of GO or RGO*x* solution (0.5 mg mL^−1^) and 0.5 mL of 1 mM EY were added into trimethylamine (TMA) solution (93.5 mL, 7.7 × 10^−2^ mol L^−1^). The mixture was stirred for 5 h at room temperature in the dark, and then centrifuged at 12000 rpm for 45 min to remove RGO*x* or GO with EY. The EY concentration of the supernatant was measured on the spectrophotometer. The adsorption amount of EY onto RGO*x* was calculated based on the concentration difference (Δ*C*) before and after the mixing.

Transient absorption decay measurements for ^3^EY^*^ were performed on a LP-920 laser flash photolysis spectrometer (Edinburgh). The excitation pulses were obtained from the unfocused second harmonic (532 nm) output of a Nd:YAG laser (Brilliant b), the probe light was provided by a Xenon short arc lamp (450 W). The measured aqueous solution was prepared as follows: 6 mL of GO or RGO*x* solution (0.5 mg mL^−1^), 0.2 mL of 10 mM EY and distilled water were added to keep the volume 100 mL, and then the mixtures were stirred for 30 min at room temperature. The dispersion was transferred to a cuvette with a cover, and then aerated for 20 min with nitrogen gas before measurements.

### Photocatalytic H_2_ evolution

Photocatalytic H_2_ evolution activity was evaluated in a similar manner as described in [18]. The photocatalytic reaction was carried out in a 190 mL Pyrex cell with a side flat window at room temperature (an efficient irradiation area of ca. 16.9 cm^2^). A high pressure Hg lamp (400 W) was used as the light source, equipped with a cutoff filter (λ ≥ 420 nm) to remove radiation below 420 nm. The IR fraction of the beam was removed by means of a cool water filter to ensure an illumination with visible light only. In a typical photocatalytic experiment, 6 mL of GO or RGO*x* solution (0.5 mg mL^−1^), 1.45 mL of 10 mM EY solution, and 0.24 mL of 1.93 mM H_2_PtCl_6_ aqueous solution were added to 92.3 mL of TMA solution. Before irradiation, the suspension of the catalyst was bubbled with N_2_ for 30 min to completely remove oxygen. Sampling was operated intermittently through the septum during experiments. The amount of photocatalytic hydrogen evolution for 2 h of irradiation was determined on a gas chromatograph (TCD, 13X molecular sieve column, N_2_ as gas carrier).

The average photon ﬂux of the incident light determined on an FGH-1 Ray virtual radiation actinometer (light spectrum: 400–700 nm) was 363 μmol m^−2^ s^−1^. The apparent quantum yield (AQY) was calculated according to the following equation.


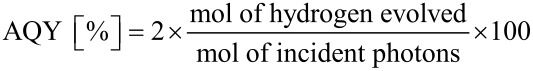


The quantum yields under monochromatic light irradiation were also measured by using various monochromatic LED lamps (UVEC-4, Shenzhen LAMPLIC Science Co.Ltd, China) as light sources. The apparent quantum yields were based on the amount of produced hydrogen for 2 h irradiation by using various LED lamps. All other reaction conditions except the light resources were identical to the conditions of the photocatalytic reaction.
